# Psychiatry

**Published:** 1904-06-04

**Authors:** 


(*To be continued.*)

### PSYCHIATRY.

**Senile Dementia.**—Senile dementia is that form of mental decay occurring after the fifth decade of life, in which the process of brain degeneration is attended with various manifestations of grave mental disorder. It is very rare before the age of 50 years in men and it is found that at least two-thirds of all patients over the age of 50 years admitted to public asylums in the United Kingdom and in America are cases of senile dementia. Pickett,[1] of Philadelphia, states that the proportion of senile dements is much higher among patients of the age of 60 years and upwards admitted to asylums. Thus out of 269 senile and insane patients over the age of 60 years 200 were cases of senile dementia—a proportion of almost exactly 75 per cent. The insanities of old age, not including senile dementia, which the remaining 69 cases exemplified were various; most of them were the terminal stages of psychoses or cerebral disorders of earlier life, such as paranoia and pre-senile delusional insanity, alcoholic and apoplectic dementia, general paralysis and epilepsy, and the vesanic and recurrent psychoses. Many of the 200 cases of true senile dementia continued free from gross dementia for a long period after admission, whereas many of the remaining 69 cases were considerably demented on admission or became so finally. Delirium in old age, says Pickett, arises quite readily, and generally it is similar in its etiology and in its clinical course to delirium in adult life. When delirium terminating life occurred in a few instances among senile patients, it was always assignable to a definite organic cause—usually nephritis, and frequently surgical or other troubles with septicæmia. Senile dementia is thus an entity clinically and etiologically separable from the various acute and chronic psychoses which (in their terminal or sub-terminal stages) may be manifested in old age; the essence of senile dementia is a quantitative change—a mental deterioration and loss, but the more obvious because obtrusive changes are of a qualitative character, viz, excitement, depression, delusion—so that the disease may appear in a guise simulating one of the vesanic psychoses, such as mania, melancholia, paranoia, etc. Senile insanities other than dementia are rare; they comprise a delusional form and a melancholic (depressive) form which belong to and are liable to occur, especially in the pre-senile period of life which corresponds with the climacteric epoch in women (from 45 to 50 years of age), or with a slightly later period (from 50 to 55 years of age) in man. Since, therefore, senile dementia is separable, clinically and etiologically, from various acute and chronic psychoses which may occur in old age "we are justified," says Dr. Pickett, "in declaring that the term 'senile insanity' is useless and even confusing." An analysis of the clinical symptoms shows that the following are the general characteristics of oncoming senile dementia: enfeeblement of attention and slowness in the association of ideas, inexact perception of the patient's relation to surroundings and to time (disorientation), marked defects of memory (amnesia) for recent events, poverty of ideas, blunting of the feelings of affection, and irritability of disposition with exacting and tyrannical tendencies. "Mental confusion of some degree is present in all types of senile dementia; it appears episodically in many (66 per cent. of) cases, being one cause of the familiar street-wandering of such dements." The excited, depressed, and delusional (paranoid) types of senile dementia rank next in order of frequency. Physical wasting and weakness are greatest in the common or confusional types of senile dementia, and are progressively less marked in the other types mentioned, a point which is of prognostic value. On the other hand the depressed and paranoid type are of less favourable prognosis as regards the mental life than are the other two types of dementia. The exultation or ephemeral excitement of some cases is not true mania; "probably true mania never arises in old age."[2] The following table gives a summary of the symptoms and the peculiarities of conduct of the 200 patients studied by Pickett. Some of the traits and habits of these patients have a medico legal importance in view of the question of responsibility for acts committed, a question which is sometimes hard to determine in such cases :—

| Symptoms and peculiarities of conduct | 143 cases of the simple confusional type | 17 cases of the excited type | 14 cases of the depressed type | 26 cases of the delusional type |
|---|---|---|---|---|
| | Per cent. | Per cent. | Per cent. | Per cent. |
| Street-wandering ... | 40 | 24 | 14 | 12 |
| Hallucinations ... | 23 | 12 | 64 | 85 |
| Vertigo ... ... | 19 | 6 | 14 | 27 |
| Headache ... ... | 8 | 6 | — | 19 |
| Night-prowling ... | 8 | 6 | 28 | 8 |
| Suicidal attempts ... | 6 | 12 | 14 | 16 |
| Exaltation ... ... | 8 | 6 | 7 | 42 |
| Delusions of poisoning | 2 | 18 | 7 | 35 |
| Delusions of conspiracy | 2 | 6 | 7 | 46 |
| Other delusions of persecution | 10 | 24 | 28 | 38 |
| Delusions of marital infidelity | — | 6 | — | 12 |

The delusional cases are an interesting and important class. Of all phases of this subject that of *fixed delusion* in the aged is the most perplexing. If we eliminate paranoiacs, alcoholic dements of the delusional type, and cases of pre-senile delusional insanity from our category of senile dementia we still have to dispose of a number of cases (26 out of 200 cases of senile dementia proper) in which delusions more or less systematised have been the troublesome feature, making the patients disagreeable, unjust, or even dangerous to their families ; though when met with in the wards of the asylum they are "uninteresting" senile dements. The chief delusions to which they are liable are those of persecution by unseen and malign agencies, poisoning, and marital infidelity, the last of which may cause much distress and trouble to the patient's family. The following two interesting cases of senile dementia are appended. Case No. 1.—The patient was a sea captain, 84 years of age, admitted under treatment in 1902. The family history was very bad, his brother had committed suicide while insane, his sister was a patient with melancholia in an asylum, and his son died insane in the Philadelphia Hospital. Two years previously the patient himself was said to be "growing childish," and had physical complaints—his "legs were going to break," he said ; then "voices" began to urge him to commit suicide ; grotesque figures and faces would appear on the walls, and he believed that his life was in great jeopardy. On admission he said (confidentially) that there was a "conspiracy against him making his life not worth a snap" if he disclosed it, and that the "Traction Company" and "U.G.I." both "secret societies" as he called them, were "putting electricity" on him. The patient talks about making a visit of revenge to those secret societies, yet he has very little idea of where he is at any time, and in all respects except for his delusions is a garrulous, foolish, senile dement. Case No. 2.—This was a woman aged 65 years, of whose family history it is only known that her father died of apoplexy. About a year before her admission in 1899 she was observed to be failing in mind and body, and to be growing over-religious. "She wandered from church to church, was several times lost in the streets, and in this manner finally reached the hospital through the police." For several months she had refused to eat any food prepared by her daughter, with whom she lived, and had tried to prevent the daughter's children from eating it "for fear of poison." She would stop strangers in the street, or policemen, and complain that her daughter and daughter's husband were going to kill the children "to get life insurance money." She one day rented a house and took the children to it where, after two days, they were found with their demented grandmother, who had not attempted even to feed them, her sole thought having been to save them from their "murderous" parents. In the hospital this patient recognised numerous patients, nurses, and doctors as her nieces and nephews, promised them houses and money, and in all her works and ways was a typical senile dement.

[1] Jour. of Nerv. and Mental Diseases, Feb. 1904. [2] Ibid.

